# Association of ALOX12-Mediated AA/12–HETE Metabolic Dysregulation with Abdominal Aortic Aneurysm Enlargement and Dissection

**DOI:** 10.3390/jcdd13090440

**Published:** 2026-09-07

**Authors:** Jianbin Xiao, Tao Qiu, Hong Wang, Hongji Zha, Jiayue Chen, Wenjia Ai, Qiang Li, Yangyong Li, Songwei Chen, Jianxiang Wang, Huan Cai, Zhihui Zhang

**Affiliations:** 1 Department of Vascular Surgery, The Second Affiliated Hospital of Guangzhou Medical University, Guangzhou 510245, China; jianbin_xiao@126.com (J.X.); 13021103939@163.com (T.Q.); zhj11451419@163.com (H.Z.); 18026029737@163.com (J.C.); think1805@126.com (W.A.); lq010709@163.com (Q.L.); lyy9801318@163.com (Y.L.); chenswmail@163.com (S.C.); 13966218682@163.com (J.W.); 13570571937@163.com (H.C.); 2 Outpatient Department Office, The Second Affiliated Hospital of Guangzhou Medical University, Guangzhou 510260, China; 13794365369@163.com

**Keywords:** aneurysm dissection, arachidonic acid metabolism, lipid metabolism, aneurysm rupture, risk factor

## Abstract

Abdominal aortic aneurysm (AAA) enlargement is a major risk factor for aneurysm rupture and the development of abdominal aneurysm dissection (AAD). However, reliable serum biomarkers for predicting AAD occurrence remain lacking. This study was designed to identify circulating serum markers capable of predicting AAA progression and AAD formation. Patients diagnosed with AAA or AAD at our institution between 2020 and 2025 were enrolled and corresponding clinical characteristics were analyzed. Peripheral blood levels of arachidonic acid (AA) and 12–HETE were measured using ELISA, and Alox12 mRNA expression was determined by quantitative real-time polymerase chain reaction (qRT-PCR). Candidate serum biomarkers associated with AAA enlargement and AAD development were investigated using a multi-modal approach integrating computed tomography (CT) images, serum metabolic profiles, and aneurysm wall tissue analyses. A total of 52 AAA patients and 42 AAA patients were included in the analysis. Elevated serum 12–HETE and TG levels were significantly associated with AAA enlargement and the occurrence of AAD, whereas AA levels showed no significant difference between groups. Dysregulated AA/12–HETE metabolism is an independent risk factor for aneurysmal enlargement in AAA and AAD, while 12–HETE and TG help to discriminate AAD from AAA.

## 1. Introduction

Abdominal aortic aneurysm (AAA) is characterized by aortic dilation with a diameter 1.5 times that of the responsible artery reaching ≥30 mm in diameter [1]. Without surgical intervention, AAA causes mortality of 60% and is detected incidentally in physical examination [2,3]. Acute aortic dissection (AAD) is characterized by acute aortic wall layer separation following an intimal tear: blood enters the intima-media space, forming an intramural hematoma that may induce aortic dilation [4,5]. Despite improvements in imaging and surgical techniques, the lack of reliable biomarkers for early diagnosis, risk stratification, and therapeutic monitoring remains a critical clinical gap. Reliable serum biomarkers for predicting aneurysm enlargement, distinguishing AAD from AAA, and guiding surgical intervention timing remain lacking [6,7]. This gap is exacerbated by the non-specific clinical manifestations of AAD, leading to frequent misdiagnosis and poor prognostic outcomes.

Lipid metabolism dysregulation, especially arachidonic acid (AA) derivatives, has emerged as a key player in vascular inflammation and remodeling [8,9]. AA, a polyunsaturated fatty acid, is metabolized to bioactive mediators via multiple pathways, with 12-Hydroxyeicosatetraenoic Acid (12–HETE) being a critical pro-inflammatory metabolite catalyzed by ALOX12 [10,11]. The metabolic intermediate 12–HETE has emerged as a key mediator, regulating macrophage homeostasis, endothelial cell death, and vascular dysfunction [12,13]. However, the role of AA/12–HETE metabolic imbalance in AAA and AAD remains unclear. Previous studies have reported that the systemic immune such as IL-6,D-dimer and CXCL17, are associated with aortic dissection [14,15,16]. However, these indicators are mainly inflammatory factors, which are themselves risk factors for AAD. Therefore, it is more important to identify more specific and upstream predictors for the occurrence of AAD.

Notably, aneurysm diameter is a well-established predictor of rupture risk [17,18], but its correlation with metabolic markers such as 12–HETE and ALOX12 expression has not been systematically investigated. Additionally, while TG accumulation induces vascular inflammation in coronary stroke and atherosclerosis [19,20], its interplay with the AA/12–HETE pathway in aortic diseases is underexplored. To address these gaps, this study enrolled AAA and AAD patients, analyzing plasma AA, 12–HETE, and TG levels, alongside ALOX12 expression in aneurysm tissues. We hypothesized that dysregulated AA/12–HETE and TG metabolism help to distinguish AAD from AAA. The findings aim to uncover novel pathogenic mechanisms and provide clinical tools for risk stratification and intervention optimization in abdominal aortic aneurysm.

## 2. Methods

### 2.1. Study Participants and Clinical Management

Patient informed consent was waived with ethical approval. Consecutive patients with AAA or AAD admitted to our institution between 2020 and 2025 were enrolled. Abdominal aortic aneurysm (AAA) and abdominal aortic dissection (AAD) are anatomically defined as lesions occurring within the abdominal aorta segment extending from the aortic hiatus to the origin of the common iliac arteries. All abdominal aortic dissection cases included in our cohort belong to Stanford type B/DeBakey type III aortic dissection. Importantly, the abdominal aortic dissections enrolled in the present study originate from a primary intimal tear directly arising in the abdominal aorta, rather than secondary dissection formed by tearing within a pre-existing abdominal aortic aneurysm. The inclusion criteria were: (1) confirmed diagnosis of AAA or AAD via computed tomography (CT) angiography; (2) availability of complete clinical records and plasma samples; and (3) no prior aortic surgery or interventional treatment. Exclusion criteria were as follows: (1) history of cancer or tumor; (2) concurrent diabetes mellitus; (3) hyperlipidemia (≥2.3 mmol/L); (4) history of renal failure; (5) unavailable CT images; (6) age under 18 years; and (7) hemolysis during ELISA testing. All patients underwent standardized clinical management.

### 2.2. Data Acquisition

Demographic and clinical characteristics were collected, including age, comorbidities (diabetes mellitus, hyperlipidemia, hypertension), and tumor history (type and diagnosis time). Aneurysm diameter was measured using CT images: the maximum diameter of AAA was identified on axial CT slices and further verified by three-dimensional reconstruction. AAA patients were stratified into three subgroups based on diameter: 30 mm ≤ Dia < 40 mm, 40 mm ≤ Dia < 50 mm, ≥50 mm; AAD patients were divided into four subgroups: <30 mm, 30 mm ≤ Dia < 40 mm, 40 mm ≤ Dia < 50 mm, ≥50 mm. TG concentration was collected from electronic medical records.

### 2.3. Measurement of AA and 12–HETE

The concentrations of AA and 12–HETE were measured via Elisa (Arachidonic Acid ELISA Kit, E-EL-0051, Elabscience, Wuhan China; 12-Hydroxyeicosatetraenoic Acid ELISA Kit, EU3131, Fintest, Wuhan, China) according to the manufactory’s instruction. Briefly, peripheral venous blood samples (5 mL) were collected from patients before treatment, anticoagulated with EDTA, and centrifuged at 3000 rpm for 10 min at 4 °C to separate plasma. Plasma samples were stored at −80 °C until ELISA detection. Standards and plasma samples were added to pre-coated wells and incubated at 37 °C for 1 h. After washing, biotinylated detection antibody was added, followed by streptavidin−HRP conjugate. Tetramethylbenzidine substrate was added for color development, and the reaction was stopped with stop solution. Absorbance was measured at 450 nm, and concentrations were calculated from the standard curve.

### 2.4. Quantitative Real-Time Polymerase Chain Reaction

Aneurysm wall tissue samples of different diameters were obtained from patients undergoing surgical resection (*n* = 6), immediately frozen in liquid nitrogen, and stored at −80 °C. Total RNA was extracted from aneurysm wall tissues using TRIzol reagent. RNA purity and concentration were verified via NanoDrop. Complementary DNA (cDNA) was synthesized with a reverse transcription kit. qRT-PCR was performed using specific primers for *ALOX12* and *GAPDH* (internal control) on a real-time PCR system. The reaction conditions included initial denaturation at 95 °C for 5 min, followed by 40 cycles of 95 °C for 10 s and 60 °C for 30 s. Relative mRNA expression was calculated using the 2^−ΔΔCt^ method. The primers of *ALOX12* and *GAPDH* were as follows:

*ALOX12:* Forward: AGCTCCTGGAACTGCCTAGA;

Reverse: AGGACAGAGGGAGCAGTAGG

*GAPDH:* Forward: AATGGGCAGCCGTTAGGAAA;

Reverse: GCGCCCAATACGACCAAATC;

### 2.5. Statistical Analysis

All data were analyzed using Prism 9.0 software. Continuous variables are presented as mean ± standard deviation. Categorical baseline variables were compared using Fisher’s exact test. The Shapiro–Wilk test was used to test the normality of continuous variables. Normally distributed data were compared with unpaired *t*-test, and non-normal data were analyzed with the Mann–Whitney U test. Differences among multiple subgroups were analyzed by one-way ANOVA for normally distributed data or Kruskal–Wallis test for datasets that failed the normality test. Receiver operating characteristic (ROC) curves were constructed to assess the diagnostic value of metabolic markers. Spearman’s rank correlation analysis was used to assess associations between AA, 12–HETE, 12–HETE/AA ratio, and TG levels. *p* < 0.05 was considered statistically significant.

## 3. Results

### 3.1. Clinical Characteristics of Patients

**Figure 1:**
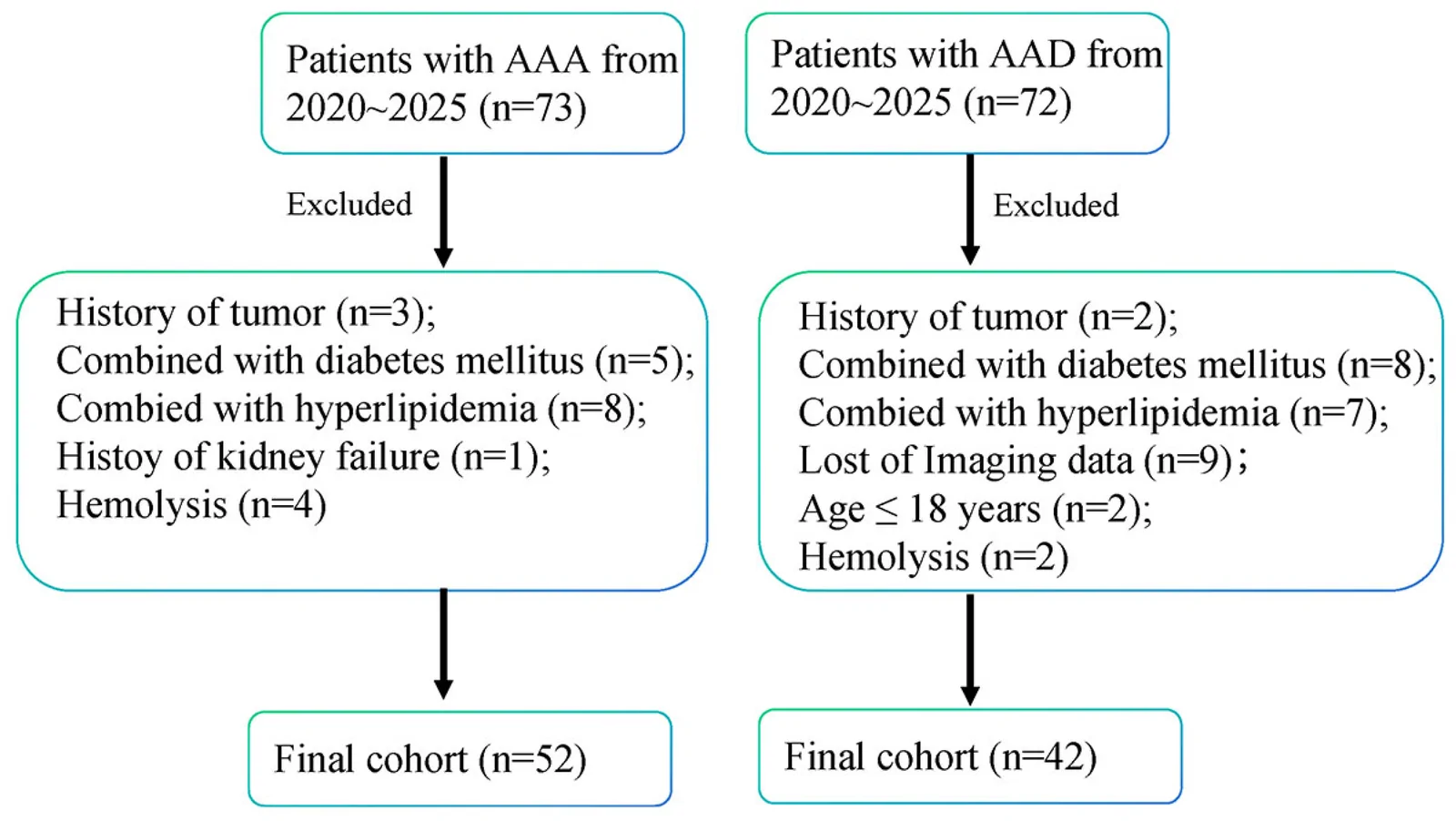
Flowchart illustrating patient enrollment and prognostic scoring in the study. AAA, abdominal aortic aneurysm; AAD, abdominal aneurysm dissection.

A total of 73 patients diagnosed with AAA and 72 patients with AAD were enrolled in this study. In patients with AAA, three had a history of tumor, one with a brain tumor and two with lung cancer; five patients had diabetes mellitus; eight patients had hyperlipidemia; and one patient had a history of kidney failure. In patients with AAD, two patients had a history of gastric cancer, eight had diabetes mellitus; seven had hyperlipidemia, nine were excluded due to loss of the CT image; two were under 18 years old; and two were excluded because of hemolysis during Elisa. Finally, a total of 52 patients with AAA and 42 patients with AAD were enrolled in this study (Figure 1). The baseline characteristics of patients involved in this study are listed in Table 1.

### 3.2. Imbalance of AA and 12–HETE Metabolism Between AAA and AAD

**Figure 2:**
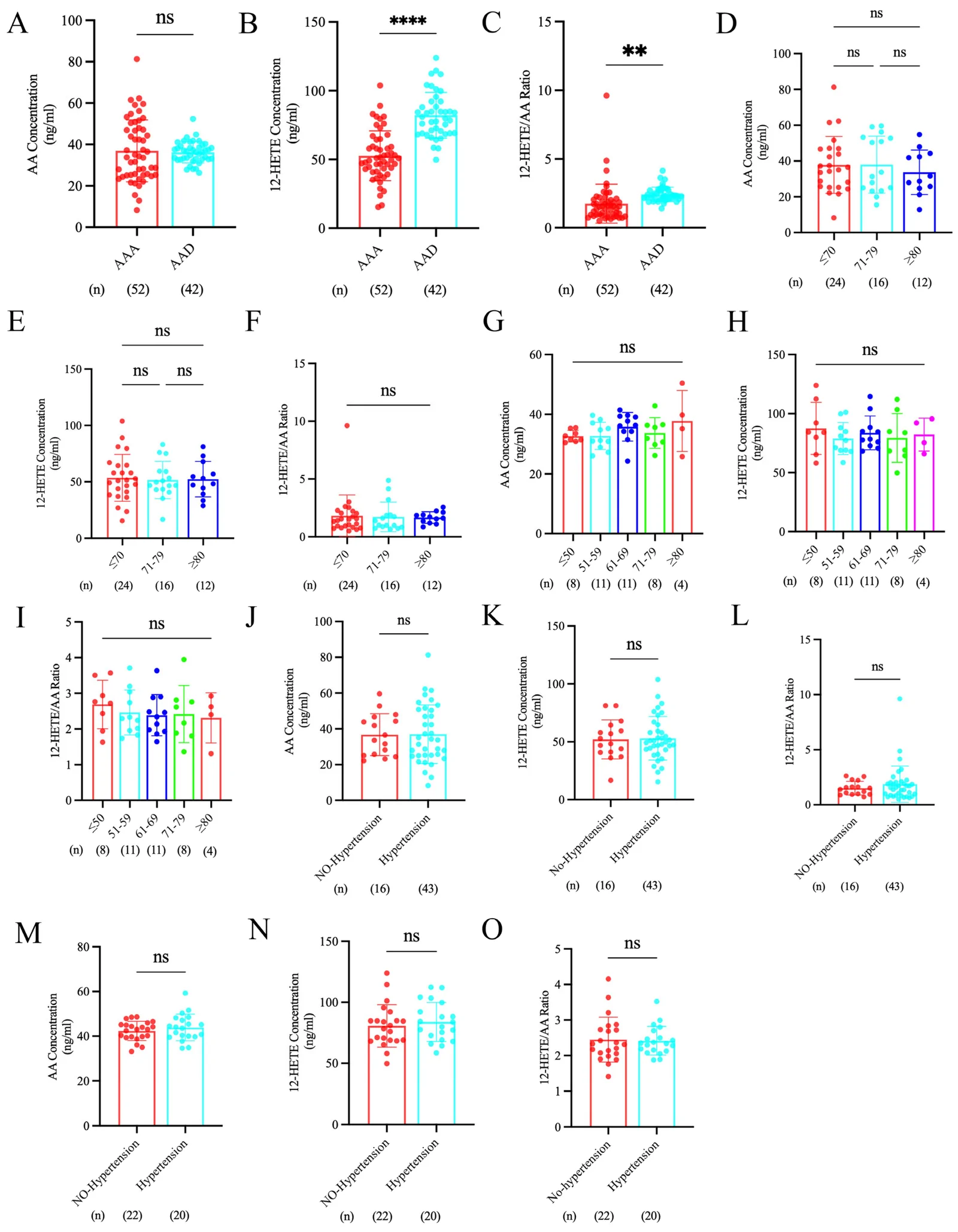
Elevated 12–HETE in AAD is not associated with blood pressure or age. (**A**) Plasma AA concentrations measured by ELISA in AAA and AAD groups. (**B**) Plasma 12–HETE concentrations measured by ELISA between AAA and AAD groups. (**C**) Changes in the 12–HETE/AA ratio between AAA and AAD groups. (**D**) Plasma AA concentration in AAA patients stratified by age. (**E**) Plasma 12–HETE concentration in AAA patients stratified by age. (**F**) 12–HETE/AA ratio in AAA patients stratified by age. (**G**) Plasma AA concentration in AAD patients stratified by age. (**H**) Plasma 12–HETE concentration in AAD patients stratified by age. (**I**) 12–HETE/AA ratio in AAD patients stratified by age. (**J**) Plasma AA concentration in AAA patients with or without hypertension. (**K**) Plasma 12–HETE concentration in AAA patients with or without hypertension. (**L**) 12–HETE/AA ratio in AAA patients with or without hypertension. (**M**) Plasma AA concentration in AAD patients with or without hypertension. (**N**) Plasma 12–HETE concentration in AAD patients with or without hypertension. (**O**) 12–HETE/AA ratio in AAD patients with or without hypertension. Statistical analysis was performed using unpaired *t*-test (**A**–**C**, **J**–**O**) and one-way ANOVA (**D**–**I**). AA, arachidonic acid; 12–HETE, 12-Hydroxyeicosatetraenoic Acid; AAA, abdominal aortic aneurysm; AAD, abdominal aneurysm dissection; ns, no significant; ** *p* < 0.01; **** *p* < 0.0001.

There was no significant difference in AA concentration in plasma between AAA and AAD (36.92 ± 14.96 ng/mL vs. 36.23 ± 5.139 ng/mL, *p* = 0.7549) (Figure 2A). However, 12–HETE was significantly higher in plasma of patients with AAD than that in those with AAA (52.70 ± 18.12 ng/mL vs. 82.22 ± 16.63 ng/mL, *p* < 0.0001) (Figure 2B). In addition, the ratio of 12–HETE to AA was also higher in patients with AAD than that in those with AAA (1.758 ± 0.246 vs. 2.433 ± 0.528, *p* = 0.0023) (Figure 2C). However, there were no significant differences in AA, 12–HETE, or 12–HETE/AA ratio among different ages and in those with or without hypertension, both in AAA and in AAD (all *p* > 0.05) (Figure 2D–O).

### 3.3. Diameter-Dependent Upregulation of 12–HETE Metabolism in AAA and AAD

**Figure 3:**
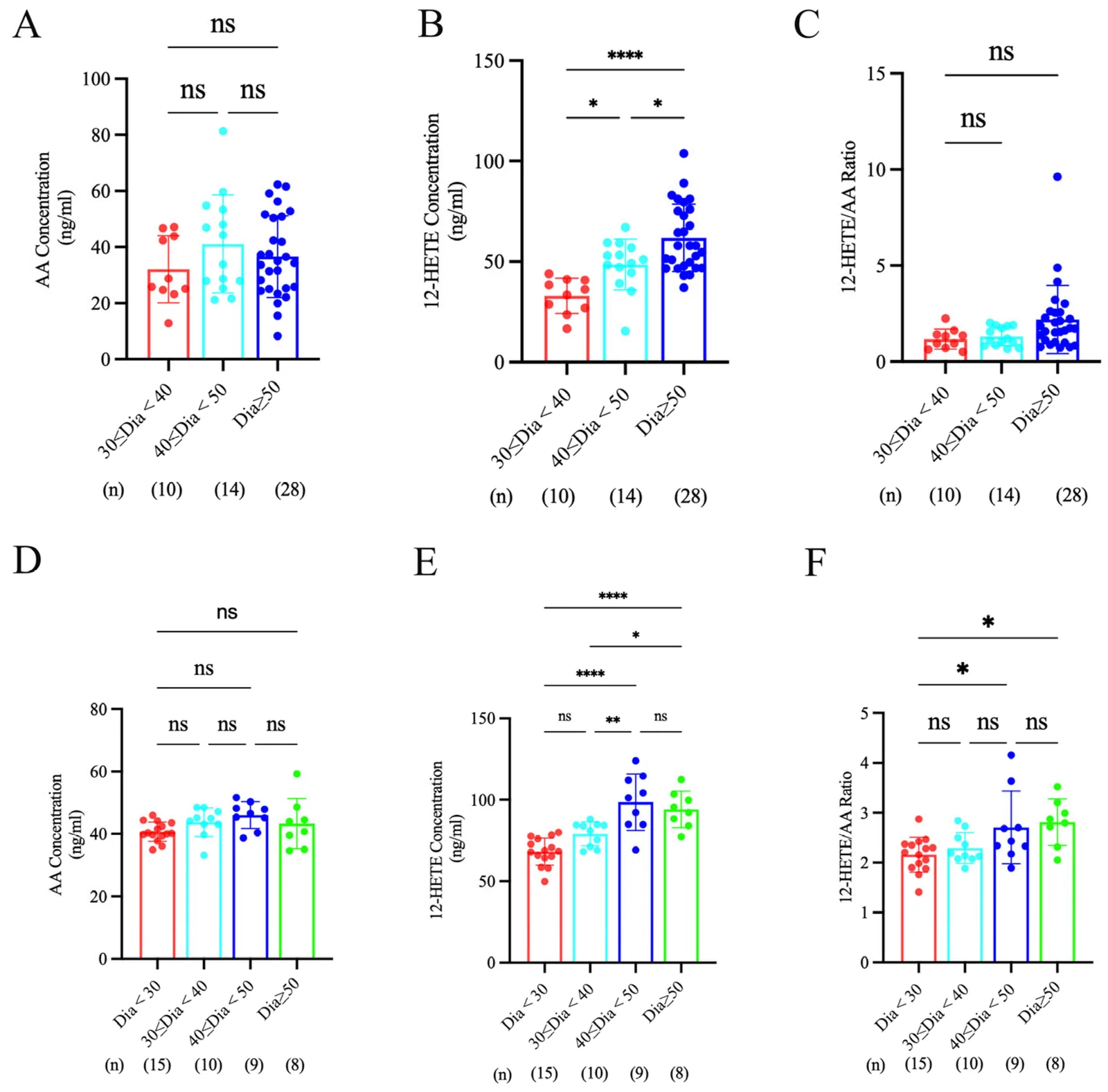
Aneurysm enlargement in AAA and AAD correlates with 12–HETE expression. (**A**,**B**) Concentration of AA divided by aneurysm diameter (mm) among different groups of AAA or AAD patients. (**C**,**D**) 12–HETE concentration divided by aneurysm diameter (mm) in groups of AAA or AAD patients. (**D**–**F**) Ratio of 12–HETE/AA divided by diameter in groups of AAA or AAD patients. AA, arachidonic acid; 12–HETE, 12-Hydroxyeicosatetraenoic Acid; Dia, diameter; ns, no significant; * *p* < 0.05; ** *p* < 0.01; **** *p* < 0.0001.

We next determined whether alterations in AA and 12–HETE levels were associated with aneurysm diameter in patients with AAA and AAD. AAA patients were stratified into three groups: ≥30 to <40 mm, ≥40 to <50 mm, and ≥50 mm. AAD patients were divided into four groups: <30 mm, ≥30 to <40 mm, ≥40 to <50 mm, and ≥50 mm. AA levels did not differ across aneurysm diameter subgroups in either AAA or AAD (*p* > 0.05) (Figure 3A,B). By contrast, 12–HETE levels increased with increasing aneurysm diameter in AAA and showed a similar rising trend in AAD (Figure 3C,D). Notably, the 12–HETE/AA ratio did not increase with aneurysm diameter in AAA (Figure 3E), whereas this ratio exhibited a progressive increase with diameter in AAD (Figure 3F).

### 3.4. ALOX12 Upregulation in AAA and AAD Drives HETE Metabolism

**Figure 4:**
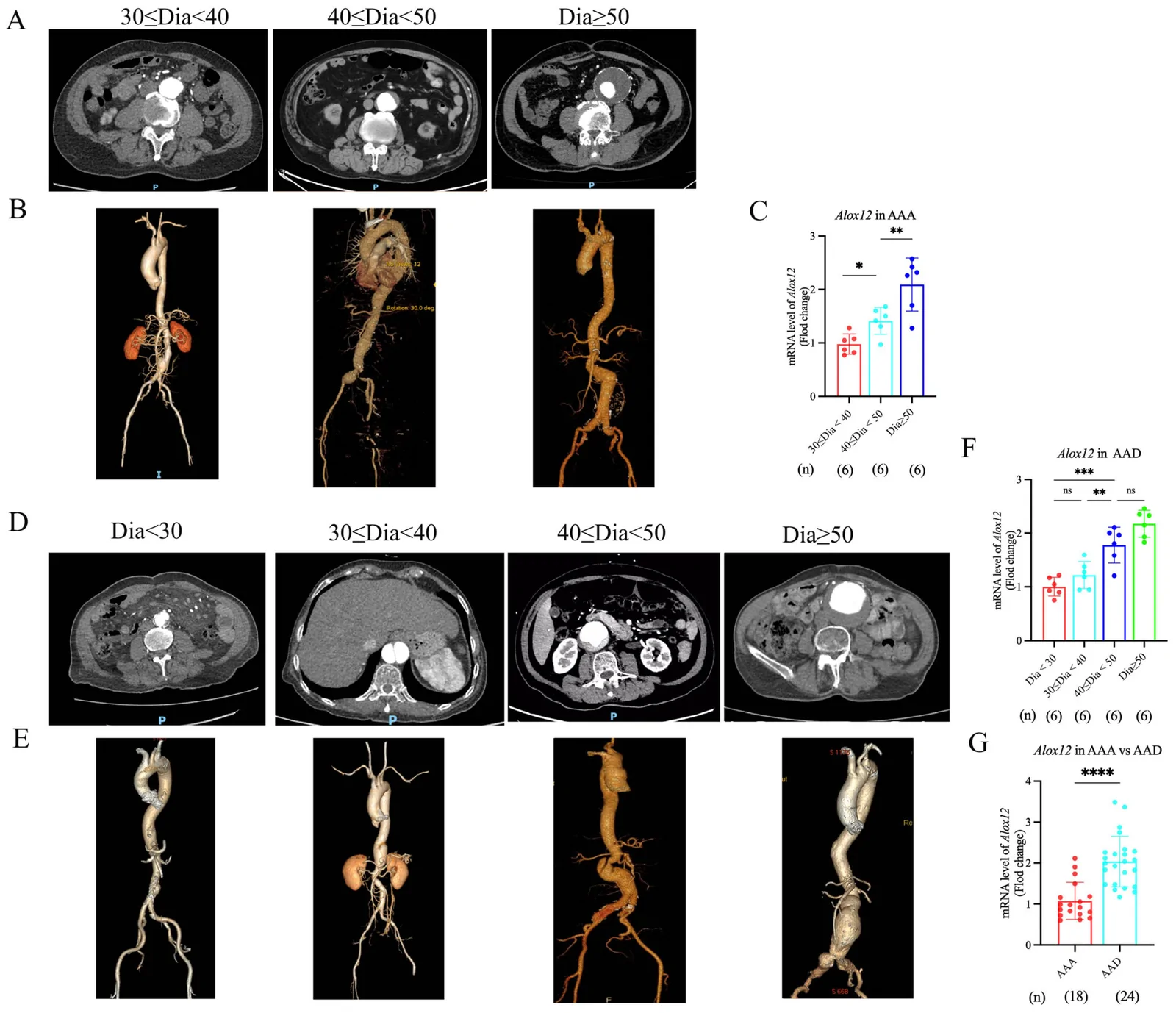
Aneurysm diameter-dependent imaging characteristics and ALOX12 expression in AAA and AAD patients. (**A**) Representative axial CT images at the level of maximal aneurysm diameter in different diameter subgroups of AAA patients. (**B**) Corresponding CTA images for the AAA subgroups shown in (**A**). (**C**) Relative mRNA expression of ALOX12 in AAA tissues from different diameter subgroups (*n* = 6). (**D**) Representative axial CT images at the level of maximal aneurysm diameter in different diameter subgroups of AAD patients. (**E**) Corresponding CTA images for the AAD subgroups shown in (**D**). (**F**) Relative mRNA expression of ALOX12 in AAD tissues from different diameter subgroups (*n* = 6). (**G**) Comparison of ALOX12 mRNA expression levels between AAA and AAD tissues (*n* = 18 in AAA, *n* = 24 in AAD). AAA, abdominal aortic aneurysm; AAD, abdominal aneurysm dissection; Dia, diameter; ns, no significant; Statistical significance is indicated by * *p* < 0.05; ** *p* < 0.01; *** *p* < 0.001; **** *p* < 0.0001.

As ALOX12 catalyzes the conversion of AA to 12–HETE [21], we measured Alox12 expression in aneurysm wall tissue. The maximum AAA diameter was identified on computed tomography (CT) images (Figure 4A), and aneurysm diameters were further confirmed by three-dimensional reconstruction (Figure 4B). Alox12 expression was then analyzed across different diameter subgroups. qRT-PCR results demonstrated that Alox12 mRNA levels were elevated with increasing aneurysm diameter in AAA (Figure 4C), and a similar pattern was observed in AAD (Figure 4D–F). In addition, Alox12 expression was significantly higher in AAD than in AAA (*p* < 0.01) (Figure 4G).

### 3.5. 12–HETE Metabolism-Associated TG Elevation Distinguishes AAD from AAA

**Figure 5:**
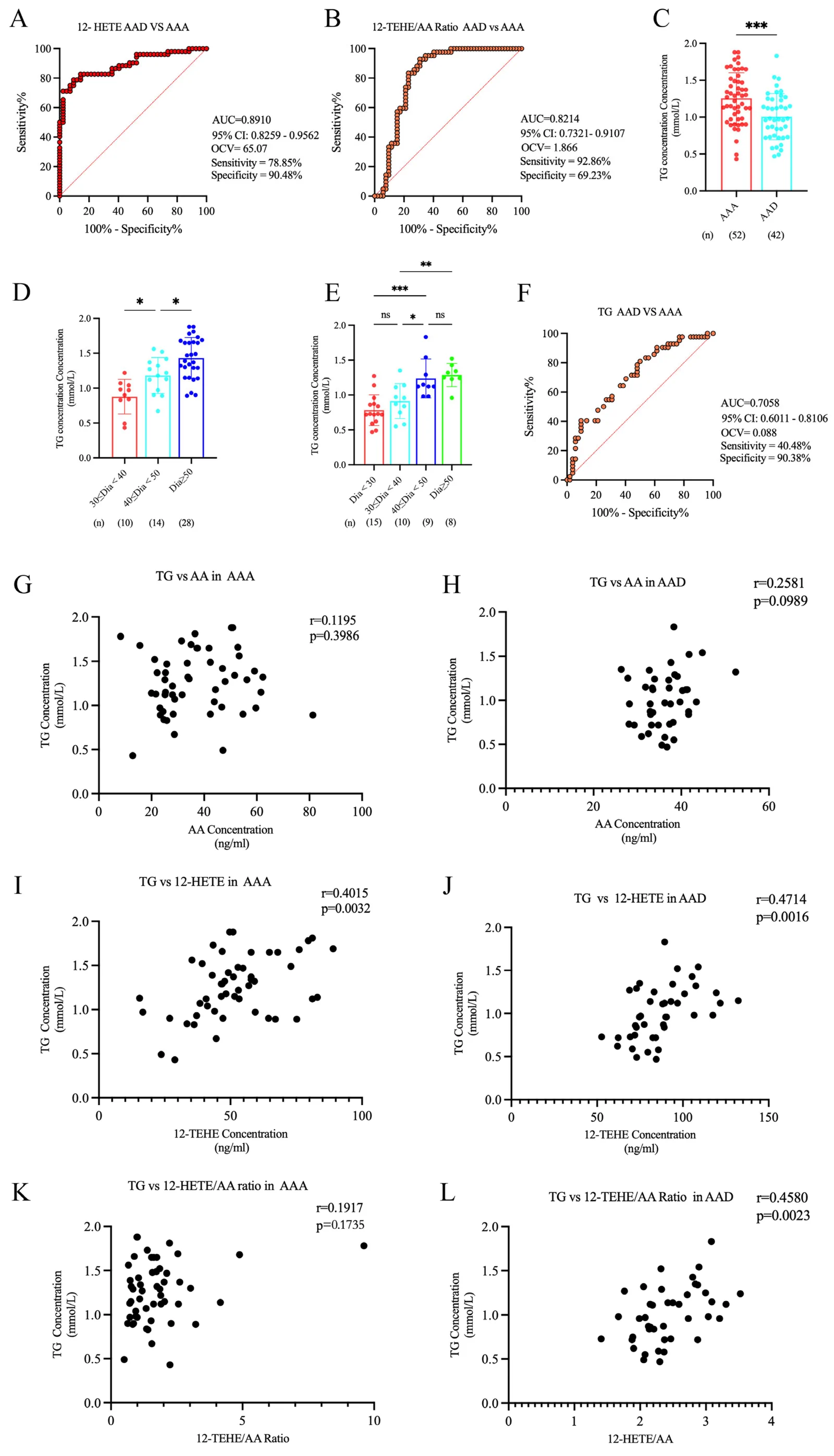
Diagnostic value of 12–HETE-related metabolites and triglyceride, and their correlations in patients with AAA and AAD. (**A**) ROC curve of 12–HETE for differentiating AAA from AAD. (**B**) ROC curve of the 12–HETE/AA ratio for differentiating AAA from AAD. (**C**) Plasma triglyceride levels in AAA and AAD groups. (**D**) Plasma triglyceride levels across different diameter subgroups in AAA patients. (**E**) Plasma triglyceride levels across different diameter subgroups in AAD patients. (**F**) ROC curve of triglyceride for differentiating AAA from AAD. (**G**,**H**) Spearman correlation analysis between triglyceride and AA in AAA and AAD. (**I**,**J**) Spearman correlation analysis between triglyceride and 12–HETE in AAA and AAD. (**K**,**L**) Spearman correlation analysis between triglyceride and the 12–HETE/AA ratio in AAA and AAD. Data were analyzed by unpaired *t*-test, one-way ANOVA, and Spearman correlation analysis. AAA, abdominal aortic aneurysm; AAD, abdominal aneurysm dissection; Dia, diameter; ns, no significant; * *p* < 0.05; ** *p* < 0.01; *** *p* < 0.001.

We next evaluated the biomarker value of 12–HETE and the 12–HETE/AA ratio for differentiating AAD from AAA. ROC curve analysis indicated that both 12–HETE and the 12–HETE/AA ratio were useful for identifying AAD. For plasma 12–HETE (Figure 5A), the area under the ROC curve (AUC) was 0.8910 (95% CI: 0.8259–0.9562), the optimal cutoff value was 65.07, and 12–HETE yielded a sensitivity of 78.85% and a specificity of 90.48%. For the 12–HETE/AA ratio, the AUC reached 0.8214 (95% CI: 0.7321–0.9107). Using the optimal cutoff value of 1.866, the biomarker achieved a sensitivity of 92.86% and a specificity of 69.23%. (Figure 5B). However, the mechanism by which dysregulated AA and 12–HETE metabolism contributes to AAA enlargement and AAD development has not been reported. We therefore hypothesized that the effects of dysregulated AA and 12–HETE metabolism—key upstream events in lipid metabolism—on AAA and AAD may be associated with TG. Accordingly, we retrospectively analyzed TG levels in the enrolled patients. TG levels differed significantly between AAA and AAD groups (Figure 5C). Moreover, TG levels increased with aneurysm expansion in both AAA and AAD groups (Figure 5D,E). The AUC value for TG was 0.7058 (95% CI: 0.6011–0.8106). At the optimal cutoff value of 0.088, TG achieved a specificity of 90.38% but only a moderate sensitivity of 40.48% (Figure 5F).

To determine whether AA affects the development of AAA and AAD via TG metabolism, correlation analyses between AA and TG were performed separately in AAA and AAD cohorts. The results showed no significant correlation between AA and TG in AAA (r = 0.1195, *p* = 0.3986) and AAD (r = 0.2581, *p* < 0.0989) (Figure 5G,H). However, TG was positively correlated with 12–HETE both in AAA and AAD (r = 0.4015, *p* = 0.0032 in AAA; r = 0.4714, *p* = 0.0016 in AAD) (Figure 5I,J). For the 12–HETE/AA ratio, no significant correlation with TG was found in AAA (r = 0.1917, *p* = 0.1735), whereas a strong positive correlation was detected in AAD (r = 0.4580, *p* = 0.0023) (Figure 5K,L).

No significant intergroup differences were observed in BMI, HDL-C, LDL-C, or total cholesterol (TCH) levels between patients with AAA and AAD or among groups with different diameters (Supplementary Figure S1). However, significant differences in TCH concentrations were detected within AAA subgroups: specifically, differences existed between the group with diameter of 30 mm ≤ D < 40 mm and the subgroup with diameter of 40 mm ≤ D < 50 mm (Supplementary Figure S1K).

## 4. Discussion

The present study investigated the AA/12–HETE metabolic pathway, ALOX12 expression, and TG levels in AAA and AAD patients, uncovering novel mechanistic insights and potential clinical biomarkers. Our findings highlight the central role of dysregulated AA/12–HETE metabolism in aneurysm enlargement dysfunction, and the utility of 12–HETE and TG for distinguishing AAD from AAA.

A key observation from this study is the selective dysregulation of 12–HETE, but not AA, in AAD compared to AAA. Plasma AA levels were comparable between the two groups, while 12–HETE concentrations and the 12–HETE/AA ratio were significantly elevated in AAD (Figure 2A). This discrepancy suggests that the metabolic flux from AA to 12–HETE, rather than AA itself, is a critical driver of disease-specific pathology. AA, as a ubiquitous polyunsaturated fatty acid, serves as a precursor for multiple bioactive lipid mediators [22], including prostaglandins and leukotrienes, which are involved in diverse inflammatory responses [23,24,25]. The unchanged AA levels across disease groups may reflect a compensatory balance in global eicosanoid metabolism, whereas the upregulation of 12–HETE points to a dominant role of the ALOX12-mediated pathway in AAD. Notably, AA, 12–HETE, and their ratio were not influenced by age or hypertension status in AAA and AAD, indicating that the observed metabolic dysregulation is intrinsic to AAA and AAD pathology rather than confounding comorbidities—a finding that strengthens the specificity of these markers.

The diameter-dependent elevation of 12–HETE in both AAA and AAD further supports its pathogenic role in aneurysm remodeling. As aneurysm is a well-established predictor of rupture risk and dissection [26,27], the progressive increase in 12–HETE with aneurysm enlargement suggests that this metabolite may amplify vascular damage. Intriguingly, the 12–HETE/AA ratio showed a diameter-dependent increase only in AAD, not in AAA (Figure 3C,F). This disease-specific pattern may reflect distinct metabolic regulatory mechanisms: in AAD, the acute surge in ALOX12 activity (as supported by higher ALOX12 expression) may overwhelm AA turnover, leading to a disproportionate increase in 12–HETE relative to AA. In contrast, AAA may involve a more chronic, balanced dysregulation where 12–HETE elevation is offset by parallel changes in AA metabolism, resulting in a stable ratio.

Our data further identify ALOX12 as one mediator of 12–HETE upregulation in AAA and AAD. ALOX12, showed diameter-dependent mRNA elevation in both AAA and AAD, with significantly higher expression in AAD (Figure 4C,F). This finding confirms that enhanced ALOX12 transcription drives 12–HETE accumulation, linking genetic regulation of lipid metabolism to aortic pathology. Together, these results establish a causal chain: ALOX12 upregulation, 12–HETE accumulation, aneurysm enlargement, with this pathway being more activated in AAD.

Another novel discovery is the utility of TG as a complementary biomarker for distinguishing AAD from AAA. TG levels differed significantly between the two diseases, increased with aneurysm diameter, and showed predictive value via ROC curve analysis—mirroring the performance of 12–HETE and the 12–HETE/AA ratio. As a core component of lipid metabolism, recent studies reported that TG accumulation contributes to AAA development and rupture and inhibits aneurysm repairment [28,29]. Here, in this study, we discovered that high TG caused aneurysm enlargement in both AAA and AAD. In addition, the differing levels of TG can help to distinguish AAD from AAA (Figure 5D). The association between 12–HETE metabolism and TG elevation suggests a functional crosstalk between these pathways: 12–HETE may promote TG synthesis or impair TG clearance in aortic tissues, or vice versa. Given that TG levels are easily measurable in clinical practice, combining TG with 12–HETE could enhance the accuracy of AAD diagnosis—particularly valuable in cases where imaging is inconclusive or delayed.

The clinical implications of these findings are substantial. Currently, AAD is often misdiagnosed due to non-specific symptoms, leading to delayed intervention and high mortality [2]. The ability of 12–HETE, the 12–HETE/AA ratio, and TG to distinguish AAD from AAA provides a panel of accessible biomarkers for rapid risk stratification. Moreover, the diameter-dependent expression of ALOX12 and 12–HETE offers potential therapeutic targets: inhibiting ALOX12 or blocking 12–HETE signaling could slow aneurysm growth and reduce AAD risk.

Despite these strengths, several limitations should be acknowledged. First, this is a single-center study with a moderate sample size (52 AAA and 42 AAD patients), which may limit the generalizability of our results. This cohort enrolled patients with atherosclerotic aortic disease and primary aortic disease with dysregulated collagen and regulatory protein turnover. Despite adjusting for baseline variables, as summarized in Table 1 (BMI, smoking, alcohol consumption, lipid profiles, and medication usage), other unmeasured baseline differences related to diverse aortic etiologies may remain. Such heterogeneity may contribute to variable risks of aortic dissection and fatal outcomes. This represents an important limitation of the present study. Furthermore, different etiologies of aortic disease may modify the activity of key amino acid degradation enzymes and indirectly confound our results. Since enzyme activity cannot be quantified in the current clinical cohort, we cannot exclude this potential confounding effect. This should be considered when interpreting this findings, and further pre-clinical work is required to clarify this issue. Larger multicenter prospective cohorts are needed to validate the biomarker performance and establish optimal cutoff values for clinical use.

Secondly, beyond ALOX-dependent HETE formation, arachidonic acid is also converted to epoxyeicosatrienoic acids (EETs) via cytochrome P450 (CYP) epoxygenases and to prostaglandins via cyclooxygenase (COX); both pathways contribute to aneurysm and dissection pathogenesis [30]. COX-2-derived prostaglandin E2 drives aortic medial inflammation, immune cell infiltration, and matrix-metalloproteinase activation, accelerating extracellular matrix degradation and aneurysm expansion [31]. While our study centers on LOX-produced 12-HETE, this pathway operates within the broader arachidonic acid metabolic network. Crosstalk between LOX, CYP-EET, and COX-prostaglandin signaling collectively establishes the aortic wall’s inflammatory-remodeling microenvironment. Published evidence confirms their functional roles in aortic diseases, rather than regarding them only as future-research topics. Additional mechanistic studies are needed to clarify interactions among these eicosanoid branches during aneurysm-dissection progression.

Thirdly, we focused on AA and 12–HETE; 5-HETE, 12–HETE, and 20-HETE are also functionally distinct arachidonic acid-derived metabolites. 5-HETE is generated via ALOX-5 and mainly mediates leukocyte chemotaxis and systemic inflammatory responses [32]. In addition, the major biological action of 20-HETE is vasoconstriction and blood pressure regulation [33], contributing to aneurysm risk indirectly through elevated systemic blood pressure, instead of directly triggering local aortic wall inflammatory remodeling. However, 12–HETE is produced by ALOX-12 within vascular resident cells, driving vascular smooth muscle phenotypic switching and macrophage activation [34,35], both risks for aneurysm development. Therefore, we prioritized 12–HETE as the key candidate in the present study, though we cannot exclude the auxiliary contributions of 5-HETE and 20-HETE in the overall disease progression.

Finally, while we observed correlations between metabolic markers and aortic diameter, causal relationships were not confirmed. Of note, this study did not determine whether increased ALOX12 expression is a primary cause or secondary event of disrupted HETE metabolism, given the intrinsic limitation of human observational samples. Genetic and epigenetic changes in AA degradation pathways may also contribute to metabolic reprogramming in aortic disease. Functional studies, such as in vitro experiments or in vivo animal models, are required to verify whether ALOX12/12–HETE dysregulation directly promotes TG accumulation and aortic enlargement.

## 5. Conclusions

This study demonstrates that dysregulated AA/12–HETE metabolism, driven by ALOX12 upregulation, is a key feature of aortic enlargement in both AAA and AAD. The selective elevation of 12–HETE and the 12–HETE/AA ratio in AAD, combined with the discriminative value of TG, provides a novel metabolic signature for distinguishing these two life-threatening diseases. These findings not only enhance our understanding of the pathogenic mechanisms underlying aortic diseases but also offer promising biomarkers and therapeutic targets for clinical application. Future studies should validate these results in larger cohorts, explore the causal relationships between metabolic dysregulation and aortic pathology, and evaluate the efficacy of targeting the ALOX12/12–HETE–TG axis in preclinical models.

## Figures and Tables

**Table 1:** The baseline characteristic of patients involved in this study.

Characteristics	AAA (*n* = 53)	AAD (*n* = 42)	*p* Value
Age (years) mean ± SEM	66 ± 1.4	62 ± 2.1	0.103
Gender (male) *n* (%)	44 (84.62%)	36 (85.71%)	>0.999
BMI	22.3 ± 0.34	22.7 ± 0.81	0.1687
Smoking history *n* (%)	11 (21.15%)	8 (19.05%)	>0.999
Alcohol history *n* (%)	6 (11.54%)	4 (9.52%)	>0.999
Hypertension *n* (%)	36 (69.23%)	20 (47.62%)	0.0337
Diabetes mellitus *n* (%)	10 (19.23%)	7 (16.67%)	0.7938
Medication usage			
Statin users	2 (3.85%)	3 (7.14%)	0.6533
Aspirin users	1 (1.92%)	2 (4.76%)	0.5845

AAA, abdominal aortic aneurysm; AAD, abdominal aneurysm dissection; BMI, body mass index.

## Data Availability

All datasets generated and analyzed in this study can be accessed from the Supplementary Materials.

## Supplementary Materials

The following supporting information can be downloaded at: https://www.mdpi.com/article/10.3390/jcdd13090440/s1, Figure S1: Association of BMI, HDL-C, LDL-C, TCH in AAA and AAD. A–C, BMI differences between AAA and AAD or among different diameters in AAA and AAD. D–F, HDL-C differences between AAA and AAD or among different diameters in AAA and AAD. G–I, LDL-C differences between AAA and AAD or among different diameters in AAA and AAD. J–L, TCH differences between AAA and AAD or among different diameters in AAA and AAD; and Raw data. This file contains the raw clinical and metabolic datasets for both the AAA and AAD patient cohorts used in this study.
