# Supplementary material for: Association of ALOX12-Mediated AA/12–HETE Metabolic Dysregulation with Abdominal Aortic Aneurysm Enlargement and Dissection
Source: J Cardiovasc Dev Dis. 2026 Sep 7;13(9):440. doi: 10.3390/jcdd13090440 (PMC13607776; doi:10.3390/jcdd13090440)
Supplement: Supplementary file 1 [file jcdd-13-00440-s001.zip › Supplementary Figure S1.pdf]

Supplementary Figure S1

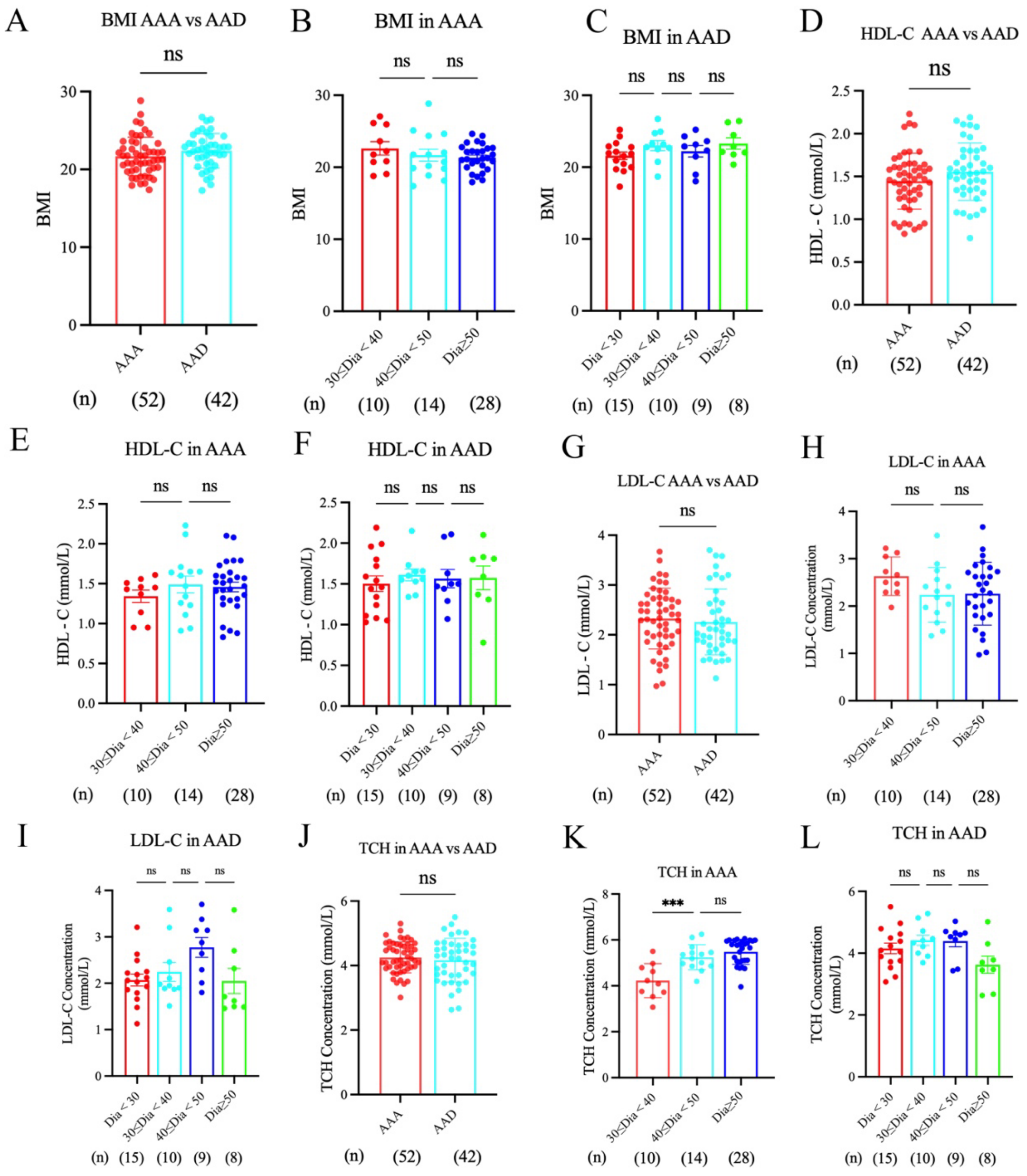

Association of BMI, HDL-C, LDL-C, TCH in AAA and AAD. A-C, BMI differences between AAA and AAD or among different diameters in AAA and AAD. D-F, HDL - C differences between AAA and AAD or among different diameters in AAA and AAD. G-I, LDL - Cdifferences between AAA and AAD or among different diameters in AAA and AAD. J-L, TCH differences between AAA and AAD or among different diameters in AAA and AAD.
